# Development of Newly Formulated Nanoalumina-/Alkasite-Based Restorative Material

**DOI:** 10.1155/2021/9944909

**Published:** 2021-07-26

**Authors:** Reham M. Abdallah, Neven S. Aref

**Affiliations:** ^1^Dental Biomaterials Department, Faculty of Dentistry, Mansoura University, Mansoura, Egypt; ^2^Dental Biomaterials Department, Faculty of Dentistry, Horus University, New Damietta, Egypt; ^3^Basic Oral and Medical Sciences Department, College of Dentistry, Qassim University, Buraydah, Qassim, Saudi Arabia

## Abstract

**Purpose:**

Nanotechnology offers considerable scope in dentistry to improve dental treatment, care, and prevention of oral diseases through the use of nanosized biomaterials. This study assessed the effect of incorporating alumina nanoparticles (Al_2_O_3_ NPa) to the recently introduced alkasite-based restorative material (Cention N) on its mechanical properties and surface topographical features.

**Materials and Methods:**

Alumina nanopowder was incorporated into the powder component of Cention N at 5 and 10% (w/w). The unblended powder was used as a control. Compressive strength was assessed using a universal testing machine. Surface microhardness and roughness were evaluated using the Vickers microhardness test and surface profilometer, respectively. Surface topography was inspected using a scanning electron microscope (SEM). Data were analyzed by ANOVA and Tukey's test (*P* < 0.05).

**Results:**

Incorporation of either 5 or 10% (w/w) Al_2_O_3_ NPa into alkasite-based restorative materials (Cention N) increased both its compressive strength and surface microhardness. This increase was significant with the use of lower concentration Al_2_O_3_ NPa (5% w/w). Meanwhile, there was an increase in surface roughness values of Cention N modified with either 5 or 10% (w/w) Al_2_O_3_ NPa. This increase was only significant in the case of 10% (w/w) Al_2_O_3_ NPa.

**Conclusion:**

Incorporation of 5% (w/w) Al_2_O_3_ NPa into the newly introduced alkasite-based restorative material (Cention N) seems to produce a promising restorative material with high compressive strength and surface hardness without adversely affecting its surface roughness properties. Thus, nanotechnology implementation into Cention N restorative material may be strongly helpful for a diversity of clinical applications.

## 1. Introduction

Various direct filling materials are available in dental markets shifting from amalgams to modern bulk-fill composites [1]. Amalgam and glass ionomer cement are considered basic filling materials. They are basic in terms of their long establishment, economical, and simplicity of use. Moreover, they are usually applied in bulk without adhesive, are self-curing, and do not need complicated dental equipment [2].

However, the drawbacks related to amalgam such as the relatively high coefficient of thermal expansion, the need for matrix band during condensation, the unesthetic appearance, and the argument concerning the safety of mercury all have a role in the emergence of tooth-colored restorative materials [3]. Similarly, glass ionomer cement possesses poor mechanical properties, limited usage (unsuitable for stress-bearing situations), and low esthetic value that led to the further development of resin-based composites [4].

Numerous improvements in direct filling materials have been made with dental composites and their accompanying adhesives in recent decades [1]. Polymeric restoratives have continued to develop into the direct restorative materials of choice mainly due to their superior esthetic characteristics [5]. Composites have been the most widely used restorative materials in dentistry in recent years, with a wide variety of applications. Yet, they are considered expensive, time-consuming, and technique sensitive [6].

Consequently, dentists searched for a real alternative to silver amalgam, glass ionomer cement, and composites that is cost-effective, a fluoride-releasing product, quick, and easy to use without the complicated equipment and offers both strength and good esthetics [7].

Cention N is an “alkasite” restorative material that marks the start of a new age of restorative dentistry, such as compomer or ormocer. It is essentially a subgroup of the composite resin [7]. It is a novel bulk-fill direct posterior restorative material. This new material uses an alkaline filler that can release acid-neutralizing ions [7].

It is self-curing with elective supplementary light curing. Cention N is radiopaque, which releases fluoride, calcium, and hydroxide ions. Due to its dual-curing option, it can be utilized as a full volume (bulk) replacement material [8]. Cention N has many advantages such as bulk placement, optimal physical/mechanical properties, better esthetics, and optional light curing [9].

The use of nanomaterials in dentistry is not only supposed to enhance the properties and functionality of dental products but also serve strides forward to the development of innovative, novel products for the beneficence of patients [10]. Nanosized materials exhibit exceptional properties according to their size. Metal and metal oxide nanoparticles have been greatly investigated due to their prospective-wide applications [11].

Aluminum oxide, commonly referred to as alumina with the chemical formula Al_2_O_3_, is a chemical compound of aluminum and oxygen with strong ionic interatomic bonding that produces its desirable material characteristics. This can exist in several crystalline phases; alpha phase alumina is the strongest and the stiffest of the oxide ceramics. The desirable characteristics of alumina, such as high hardness, excellent dielectric properties, and good thermal properties, make it the material of choice for a variety of applications. Moreover, it has excellent size and shape capabilities with high strength and stiffness too [12].

As the use of nanoparticles has become a significant area of research in the dental field, the purpose of this study is to evaluate the effect of incorporating the recently introduced alkasite restorative material, Cention N with alumina nanoparticles on its compressive strength, surface roughness, and microhardness and surface microstructure.

According to the research hypothesis, adding Al_2_O_3_ NPa to Cention N would change its physical properties and surface microstructure.

## 2. Materials and Methods

A commercially available Cention N restorative powder (Cention, Ivoclar Vivadent AG, Liechtenstein, Lot Number X46009) was blended in various proportions with alumina nanoparticles (Sigma-Aldrich Co., St. Louis, MO, USA) with particle size measuring <50 nm by transmission electron microscope (TEM).

### 2.1. Specimen Preparation

Specimens' powders were made by blending 5% and 10% (w/w) alumina nanoparticles powder with the Cention N powder (with a particle size of 90 µm as received by the manufacturer) by hand using a mortar and pestle for 10 min. The unblended powder was used as the control for all tests. The recommended powder/liquid (P/L) ratio of 1.8/1 for Cention N restorative material was used in all prepared specimens. The 5 and 10% w/w of alumina NPa powder ratios were added to the Cention N powder before proportioning the powder with the liquid; hence, the additional alumina powder ratios were accompanied by the reduction in the amount of Cention N powder.

A total of 93 specimens were used in the study: 30 specimens for each mechanical test (compressive strength, surface microhardness, and surface roughness tests) and 3 representative samples, one for each of the following groups for scanning the surface microstructure.

In each test, specimens were equally divided into three groups (10 specimens each): (I) Cention N (control) prepared from the conventional Cention N powder, (II) 5% (w/w) Al_2_O_3_-NPa-modified Cention N, and (III) 10% (w/w) Al_2_O_3_-NPa-modified Cention N.

A sectional Teflon mold (8 mm diameter × 2 mm thickness) was utilized to fabricate disc-shaped specimens used for surface microhardness, surface roughness, and color stability tests. At the same time, a stainless-steel split mold (4 mm in diameter and 6 mm in height) according to ISO standards was utilized to prepare cylindrical specimens for compressive strength testing. All specimens were stored in deionized water at 37 ± 1°C to equilibrate for 48 hours before testing.

### 2.2. Compressive Strength Test

Compressive strength testing (Cs; MPa) was performed using the universal testing machine at a crosshead speed of 0.5 mm/min. It was calculated using the following equation:

(1) $$\begin{array}{c} \text{Cs}=\frac{4P_{f}}{\pi D^{2}}, \end{array}$$

where *P*_*f*_ is the load (N) at the fracture and *D* is the diameter of the specimen (mm) [13].

### 2.3. Surface Microhardness Test

The Vickers hardness numbers (VHN) for the tested specimens were obtained using a microindentation tester (MMT-3 Digital Hardness Tester, Buehler Ltd., Lake Bluff, IL) by applying a load of 29.42 N on the specimens for 30 seconds. Five indentation measurements were carried out and averaged for each specimen [14].

### 2.4. Surface Roughness Test

Using a surface profilometer (Surftest 211, Mitutoyo, Tokyo, Japan), the surface roughness of each specimen was explored in five distinct locations. The surface roughness cutoff value was 0.8 mm, and the stylus' traversing range was 4 mm. The tracing diamond tip radius was 5 *μ*m, and the measuring strength and velocity were 4 mN (0.4 g) and 0.5 m s^−1^, respectively. Each specimen shows the average roughness value (Ra, *μ*m) as the mean of the Ra values measured in five distinct locations.

### 2.5. Scanning Electron Microscopy (SEM)

The surface microstructure of the three samples representing the studied groups was examined using a scanning electron microscope (SEM; JEOL, JSM-6510LV, Japan) operating with an accelerating potential of 30 kV and magnification up to ×10^6^. All specimens were coated with a thin layer of gold to minimize the effect of charge.

## 3. Results

### 3.1. Compressive Strength

The mean and standard deviation values for compressive strength are presented in Table 1. The 5% (w/w) Al_2_O_3_-NPa-modified Cention N group showed the highest compressive strength value (202.680 ± 7.558), while the control group (no addition) showed the least value (173.787 ± 3.302). One-way ANOVA identified significant differences between the mean values of compressive strength of the tested groups (*P*=0.0012). Tukey's test showed that there was no statistically significant increase in compressive strength value of 10% (w/w) Al_2_O_3_-NPa-modified Cention N in comparison to the control group. On the other hand, there was a significant increase in compressive strength values (*P* < 0.05) of 5% (w/w) Al_2_O_3_-NPa-modified Cention N when compared to both the 10% (w/w) and the control groups.

### 3.2. Surface Microhardness

The mean and standard deviation values for surface hardness are presented in Table 1. The 5% (w/w) Al_2_O_3_-NPa-modified Cention N group showed the highest surface microhardness value (76.067 ± 2.682), while the control group exhibited the least value (48.333 ± 2.645). One-way ANOVA identified significant differences between the mean values of surface microhardness of the tested groups (*P*=0.0001). Both 5 and 10% (w/w) groups showed a significant increase in surface microhardness values when compared to the control group. The addition of a lower concentration of Al_2_O_3_ NPa (5% w/w) to Cention N significantly increased its microhardness values when compared to those of the higher concentration (10% w/w) group.

### 3.3. Surface Roughness

The mean and standard deviation values for surface roughness are presented in Table 1. The higher concentration of the Al_2_O_3_ NPa group (10% (w/w)) demonstrated the highest surface roughness value (0.1790 ± 0.0118), while the control group exhibited the least value (0.1064 ± 0.0357).

One-way ANOVA showed significant differences between the mean values of surface roughness of the tested groups (*P*=0.0003). The surface roughness value of the 5% (w/w) group exhibited a slight nonsignificant increase in comparison with that of the control group. However, the surface roughness value of the 10% (w/w) group exhibited a significant increase when compared to both the 5% (w/w) and the control groups.

### 3.4. Scanning Electron Microscopy

**Figure 1:**
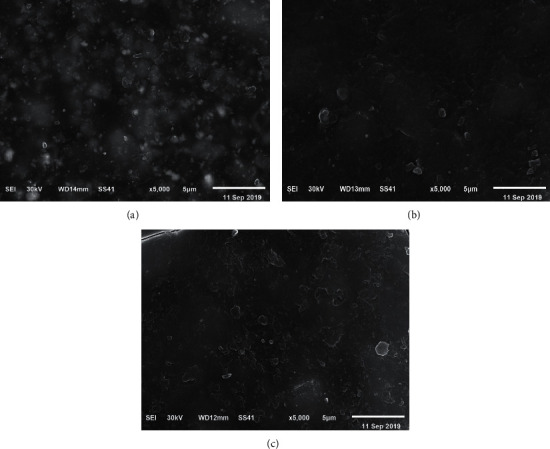
Scanning electron microscopy photographs of samples: (a) pure conventional Cention N (control), (b) Cention N at 5% (w/w) Al_2_O_3_ NPa, and (c) Cention N at 10% (w/w) Al_2_O_3_ NPa.

The SEM photomicrographs obtained in this study demonstrated an increase in the homogeneity and smoothness of the surface with modification of the Cention N samples with 5% (w/w) NPa (Figure 1(b)) in comparison to Figure 1(a). Meanwhile, the higher concentration (10% w/w) exhibited the appearance of small clusters due to the agglomeration of the powder of nanoparticles (Figure 1(c)).

## 4. Discussion

Nanomaterials are expected to enhance not only the properties and use of dental products but also the development of new products for the best benefit of patients [10]. The use of nanoscale materials, especially metal oxide nanoparticles such as Al_2_O_3_ NPa, has been investigated in this study because of their potential for a variety of applications due to their specific properties [11].

Compressive strength has a particularly important role in the mastication process since most of the masticatory forces are compressive [15]. Therefore, it is important to investigate whether the compressive force contributes to fracture failure during the mastication process. The microhardness test is a parameter frequently used to evaluate the material surface's resistance to plastic deformation by penetration [16].

The research hypothesis was accepted since the addition of Al_2_O_3_ NPa to Cention N did alter its physical properties. The two concentrations of Al_2_O_3_ NPa (5 and 10% w/w) increased the compressive strength of Cention N. However, this increase was only significant in the case of lower concentration (5% w/w). Similarly, a significant improvement in surface hardness values was exhibited by the two groups of Cention N modified with both 5 and 10% (w/w) Al_2_O_3_ NPa, which was more pronounced also with the lower concentration (5% w/w).

Compressive strength and surface hardness improvement of Cention N containing 5% and 10% (w/w) Al_2_O_3_ NPa can be attributed to the small size of the Al_2_O_3_ particles supplemented into the glass fillers of the powder. These nanoparticles could occupy the empty spaces between the larger Cention N glass filler particles and act as additional binding sites for the organic monomer part of Cention N that was found in the Cention N liquid [17]. This monomer consists of four different dimethacrylates: urethane dimethacrylate (UDMA), tricyclodecane-dimethanol dimethacrylate (DCP), tetramethyl-xylylen-diurethane dimethacrylate (aromatic aliphatic UDMA), and polyethylene glycol-400 dimethacrylate (PEG-400 DMA) that interconnect (cross-links) during polymerization resulting in strong mechanical properties and good long-term stability [18].

The lower compressive strength and surface hardness values at higher Al_2_O_3_ NPa concentration (10% w/w) loading compared to lower concentration (5% w/w) loading could be related to the Al_2_O_3_ NPa's propensity to agglomerate within the matrix at higher concentration exhibiting weak matrix interaction, resulting in lower mechanical properties [19]. Furthermore, these clumped particles may serve as a defect center, promoting the accumulation of stress-related damage [20].

This was supported by Schulze et al. who concluded that an increase in filler fraction does not necessarily lead to an increase in strength. This could be attributed to the fact that higher filler fractions could generate more defects that weaken the materials [21].

The findings of this study are consistent with Adachi et al. who reported that the addition of fillers in the form of alumina nanoparticles into a polymer that serves as a matrix improved the mechanical behavior of the obtained composite material [22].

On the contrary, the main problems encountered with the addition of higher concentrations of nanoparticles are the mixing and uniform distribution of the nanoparticles within the matrix material because nanoparticles tend to agglomerate, thus weakening the polymer matrix [21].

In the present study, the values of average surface roughness (Ra) for all tested Cention N specimens (control and modified groups) were within the 0·106–0·179 *μ*m range. Uppal et al. [23] reported that the critical surface roughness value for bacterial colonization is 0.2 *μ*m. Surface roughness higher than 0.2 *μ*m is likely to increase significantly bacterial adhesion, dental plaque maturation, and acidity, which act on material surfaces, thus increasing caries risk. In this study, all Cention N presented surface roughness below this value, both before and after modification with Al_2_O_3_ NPa.

The results of this study exhibited an increase in surface roughness values of Cention N modified with either 5 or 10% (w/w) Al_2_O_3_ NPa. However, this increase was only significant in the case of the higher concentration group (10% w/w). This might be attributed to the increasing possibility of agglomeration of Al_2_O_3_ NPa in the case of using higher concentration with the corresponding lack of homogeneity and interfacial bonding between the particles and polymer matrix and hence an accompanying increase in surface roughness.

The SEM examination of the samples in this study was consistent with the roughness results since the SEM photomicrograph of Figure 1(c) revealed the appearance of small clustering with a higher concentration of the Al_2_O_3_ NPa group when compared to those of both the control group (Figure 1(a)) and the lower concentration group of Al_2_O_3_ NPa (Figure 1(b)). This clustering tends to decrease the homogeneity of the surface of the samples [24].

The lack of water sorption and solubility tests, as well as the use of only two concentration groups of alumina NPa, added to Cention N, are regarded as limitations of this study.

## 5. Conclusions

Based on the results and within the limitations of this study, it could be concluded that the use of 5% (w/w) Al_2_O_3_-NPa-modified Cention N appears to be very promising. Modification of Cention N with 5% (w/w) Al_2_O_3_ NPa improved both compressive strength and surface hardness without compromising its surface roughness. Further assessments are demanded to study the effect of this modification on certain properties such as color change as well as water sorption and solubility.

## Figures and Tables

**Table 1:** Means and standard deviations (SD) of physical properties of Cention N with alumina NPs incorporation and Tukey's analysis.

Group	Compressive strength (MPa)		Surface microhardness (kg/mm^2^)		Surface roughness (*μ*m)	
	Mean	SD	Mean	SD	Mean	SD
Cention N (control)	173.787^b^	3.302	48.333^c^	2.645	0.1064^b^	0.0357
Cention N at 5% (w/w) alumina NPa	202.680^a^	7.558	76.067^a^	2.682	0.1138^b^	0.0026
Cention N at 10% (w/w) alumina NPa	181.753^b^	3.477	66.583^b^	2.115	0.1790^a^	0.0118

*P* value	0.0012		0.0001		0.0003	

^∗^Mean values for each property represented with the same superscript letter (column) are not significantly different (*P* ≥ 0.05), While the mean values with different letters are significantly different (*P* < 0.05).

## Data Availability

The SPSS data file used to support the findings of this study are available from the corresponding author upon request.
